# Multiperiod Maximum Loss is time unit invariant

**DOI:** 10.1186/s40064-016-2959-x

**Published:** 2016-08-11

**Authors:** Raimund M. Kovacevic, Thomas Breuer

**Affiliations:** 1 Vienna University of Technology, Wiedner Hauptstraße 8/E105-4, 1040 Vienna, Austria; 2 FH Vorarlberg, Hochschulstraße 1, 6850 Dornbirn, Austria

**Keywords:** Multiperiod risk measures, Relative entropy, Ambiguity aversion, 62C20, 90B50, 91B30, 94A17

## Abstract

Time unit invariance is introduced as an additional requirement for multiperiod risk measures: for a constant portfolio under an i.i.d. risk factor process, the multiperiod risk should equal the one period risk of the aggregated loss, for an appropriate choice of parameters and independent of the portfolio and its distribution. Multiperiod Maximum Loss over a sequence of Kullback–Leibler balls is time unit invariant. This is also the case for the entropic risk measure. On the other hand, multiperiod Value at Risk and multiperiod Expected Shortfall are not time unit invariant.

## Background

Risk measures for stochastic processes have been a popular research topic over the last decade. In this literature, time consistency has been an important property required for multiperiod risk measures on top of the properties of one period risk measures. Given a sequence of one period (conditional) risk measures, a time consistent multiperiod risk measure can be constructed easily in a recursive way:

Consider a stochastic loss process $\left\{ L_{t}:\,t\in \{1,\dots ,T\}\right\}$ defined on a filtered probability space $\left\{ \Omega ,{\mathcal {F}},\left\{ {\mathcal {F}}_{t}\right\} _{t\in \left\{ 1,\dots ,T\right\} },{\mathbb {P}}\right\}$ and a sequence $\mathrm{R}_{\alpha _{1}}, \ldots , \mathrm{R}_{\alpha _{T}}$ of one period risk measures (not necessarily coherent) with parameters $\alpha _{t}$, such that $\mathrm{R}_{\alpha _t}$ maps ${\mathcal {F}}_t$-measurable random variables to ${\mathcal {F}}_{t-1}$ measurable random variables (expressing conditional risk). As an example, parameters would be confidence levels when $\mathrm{R}$ is Value at Risk (VaR) or Expected Shortfall. We assume that ${\mathcal {F}}_0$ is the trivial $\sigma$-algebra, i.e. ${\mathcal {F}}_0=\left\{ \Omega ,\emptyset \right\}$. In this setup one may define the multiperiod risk $\mathrm{MR}_{\alpha _{1},\ldots ,\alpha _{T}}$ of the process $L_t$ recursively by

1 $$\begin{aligned} {\mathrm{MR}}_{\alpha _{T}}\left( L_{T}|\mathcal{{F}}_{T-1}\right)& := {\mathrm{R}}_{\alpha _{T}} \left( L_{T}|\mathcal{{F}}_{T-1}\right) , \nonumber \\ \mathrm{MR}_{\alpha _{t},\ldots ,\alpha _{T}}\left( L_{t},\ldots ,L_{T}|\mathcal{{F}}_{t-1}\right)& := {\mathrm{R}}_{\alpha _{t}}\left( L_{t} +\mathrm{MR}_{\alpha _{t+1}, \ldots ,\alpha _{T}}\left( L_{t+1},\ldots ,L_{T}|\mathcal{{F}}_{t-1}\right) \right) . \end{aligned}$$

Under weak technical assumptions this construction guarantees that $\mathrm{MR}_{\alpha _{1},\ldots ,\alpha _{T}}$ is time consistent, see Cheridito et al. (2006) and Kovacevic and Pflug (2009, [Proposition 3.3.5]).

In this paper we introduce *time unit invariance* as another requirement for time consistent process risk measures: If one calculates the risk of an i.i.d. process of losses that add to a total loss recursively (using 1) with identical parameters $\alpha _t=\alpha$) and compares with the one period risk of the sum, the result is the same, if the parameter for the whole time horizon is chosen appropriately. If the correct parameter value does not depend on the underlying loss distribution, we say that the risk measure is time unit invariant. We do not think risk factor changes are necessarily i.i.d. in applications. But if this were the case, a multiperiod risk of the loss process should be equal to one-period risk of aggregated losses, whatever the portfolio happens to be.

More formally:

### **Definition 1**

We call a time consistent multiperiod risk measure $\mathrm{MR}$, where MRis defined as in (1), time unit invariant if there exists a parameter value $\bar{\alpha }$, such that for any iid sequence of losses $L_1, \ldots , L_T$

2 $$\begin{aligned} \mathrm{MR}_{\alpha ,\ldots ,\alpha }\left( L_1, \ldots , L_T | \mathcal{{F}}_0\right) = R_{\bar{\alpha }}\left( \sum _{t=1}^{T}L_{t}|\mathcal{{F}}_{0}\right) \end{aligned}$$

holds, and the parameter $\bar{\alpha }$ is independent of the distribution of $L_t$.

The intuition behind requiring the same $\bar{\alpha }$ for all loss processes is that the passage of time does not depend on the portfolio someone may hold.

We analyze time unit invariance for time consistent multiperiod risk measures, based on several underlying one period risk measures, namely value at risk, expected shortfall, entropic risk measures and Maximum Loss. The main emphasis is on the introduction and analysis of a time consistent multiperiod extension of Maximum Loss, which is a one-period risk measure analyzed in Breuer and Csiszár (2013), see also Breuer and Csiszár (2010), Breuer and Csiszár (2016) and Kovacevic (2011).

The paper is structured in the following way: In “Multiperiod Maximum Loss” section we recapitulate basic facts about Maximum Loss and introduce the multiperiod version of Maximum Loss. “Main results” section proves the main results about time unit invariance of Maximum Loss. In “Examples and counterexamples” section we give an example with multiperiod Maximum Loss applied to linear and quadratic portfolios and analyze time unit invariance for further risk measures. Finally, “Conclusions” section summarizes the findings.

## Multiperiod Maximum Loss

In the single period case consider a measurable space $\left( \Omega ,{\mathcal {F}}\right)$, a random vector ${\mathbf {r}}(\cdot ): \Omega \rightarrow {\mathbb {R}}^k$, representing risk factors, and a measurable real-valued loss function $L(\cdot )$. Conditions on *L* will be specified below. We may shortly write $L=L({\mathbf {r}})$, or even *L* when the dependence on *r* is not in the foreground.

The true probability measure ${\mathcal {P}}$ is not known, but it is assumed that an estimated measure, *P*, is available. Furthermore, in order to account for model uncertainty it is assumed that the true probability measure lies within the “ball” of all probability measures *Q* whose *I*-divergence (also called relative entropy or Kullback–Leibler divergence)

3 $$\begin{aligned} D(Q||P):=\int \log \frac{dQ}{dP}({\mathbf {r}})dQ({\mathbf {r}}) \end{aligned}$$

from *P* is not larger than some fixed threshold $k>0$.

Relative entropy appears to be a versatile measure of divergence for distributions, with many applications in statistics, information theory, statistical physics see e.g. Kullback (1959), Csiszár and Körner (1981), Cover and Thomas (2006), Jaynes (1968, 1982). Moreover, relative entropy balls are a popular choice for describing model uncertainty in portfolio selection, asset pricing, and contingent claim pricing, see e.g. Friedman (2002a, b), Calafiore (2007), Barillas et al. (2009), Hansen and Sargent (2008), and others cited there.

Maximum Loss of the loss function *L* (introduced and analyzed in Breuer and Csiszár (2013), see also Kovacevic (2011)) then is defined as the expected loss in the worst of the plausible distributions *Q* with density $Z=\frac{dQ}{dP}$, i.e.

4 $$\begin{aligned} \mathrm{ML}_{k}(L)& := \sup _{Q:D(Q||P)\le k}\mathbb {E}_{Q}(L) \nonumber \\& = \sup \left\{ \mathbb {E}_{P}\left[ LZ\right] :\; Z\ge 0,\mathbb {E}_{P}\left[ Z\right] =1,\mathbb {E}_{P}\left[ Z\log \left( Z\right) \right] \le k\right\} . \end{aligned}$$

Note that

5 $$\begin{aligned} {\mathbb {E}}_{P}\left[ Z\,\log \left( Z\right) \right]& = \int \frac{dQ}{dP}\log \left( \frac{dQ}{dP}\right) dP \nonumber \\= & \int \log \left( \frac{dQ}{dP}\right) dQ\nonumber \\ & = D(Q||P). \end{aligned}$$

Maximum Loss is a coherent risk measure (see Artzner et al. 1999; Föllmer and Schied 2004) and a decision maker, trying to minimize it, is ambiguity averse in the sense of Gilboa and Schmeidler (1989). Special instances of Maximum Loss have been used already in Friedman (2002a) and Hansen and Sargent (2008), who considered linear and quadratic portfolios depending on normally distributed risk factors.

Loss *L* is not assumed to be essentially bounded. Instead, given *k* in (4) we require *L* to satisfy conditions (i–iii) below. Maximum Loss is different from the entropic risk measure (Föllmer and Schied 2004), which describes divergence preferences (Maccheroni et al. 2006), and whose dual representation also uses *I*-divergence, but as a penalty term. Still the two can be evaluated with the same techniques, see Breuer and Csiszár (2016).

The loss maximisation problem occuring in the definition (4) of $\mathrm{MaxLoss}$ has a regular solution when *L* and *k* meet three conditions (see Breuer and Csiszár 2013):

(i) If $\mathrm{ess \, sup}(L)$ is finite, then *k* should be smaller than $k_{\max }:=-\log (P(\{{\mathbf {r}}:L({\mathbf {r}})=\mathrm{ess \, sup}(L)\})),$
(ii) ${\theta _\mathrm{max}}(L):=\sup \{\theta :\Lambda (\theta )<+\infty \}$ should be positive,
(iii) If ${\theta _\mathrm{max}},\Lambda ({\theta _\mathrm{max}})$, $\Lambda '({\theta _\mathrm{max}})$ are finite, then *k* should be smaller than $k_{\max }(L):= {\theta _\mathrm{max}}\Lambda '({\theta _\mathrm{max}})-\Lambda ({\theta _\mathrm{max}})$.

Here the function $\Lambda$, in fact the cumulant generating function of the loss distribution, is defined as

6 $$\begin{aligned} \Lambda (\theta ,L):=\log \left( \int e^{\theta L({\mathbf {r}})}dP({\mathbf {r}})\right) , \end{aligned}$$

where $\theta$ is a positive real number. For fixed *L*, it can be shown that $\Lambda$ as a function of $\theta$ is convex and lower semicontinuous on $\mathbb {R}$. Its essential domain of definition $D_\Lambda :=\{\theta : \Lambda (\theta ) < \infty \}$ is a finite or infinite interval, excluding the trivial case $D_\Lambda =\{0\}$. In the interval $D_\Lambda$, the function $\Lambda (\theta )$ is continuous and has derivative $\Lambda '(\theta )=\int L({\mathbf {r}})\exp (\theta L({\mathbf {r}})-\Lambda (\theta ))dP({\mathbf {r}})$. At an endpoint of $D_\Lambda$ that belongs to $D_\Lambda$, this derivative is understood as one-sided and is not necessarily finite. Moreover, $\Lambda '(\theta )$ is strictly increasing in $D_\Lambda$ (unless $L({\mathbf {r}})$ is constant *P*-almost surely). If $\sup D_\Lambda =\infty$ then $\Lambda '(\theta )\rightarrow \mathrm{ess \, sup}(L)$ as $\theta \rightarrow \infty$.

Under assumptions (i), (ii), (iii) the equation

7 $$\begin{aligned} \theta \Lambda '(\theta ,L)-\Lambda (\theta ,L)=k, \end{aligned}$$

where $\Lambda '(\theta ,L)$ denotes the derivative w.r.t. $\theta$, has a unique positive solution ${\overline{\theta }}$. The Maximum Loss then is achieved and is given by

8 $$\begin{aligned} \mathrm{ML}_{k}(L)=\Lambda '({\overline{\theta }},L). \end{aligned}$$

The pathological cases where some of the assumptions (i–iii) are violated can be solved with different methods, see Breuer and Csiszár (2013). In the sequel (i–iii) are standing assumptions.

Extending (4), conditional versions of one-period Maximum Loss can easily be introduced. Given some $\sigma$-field $\mathcal{{F}}'\subset \mathcal{{F}}$, define the conditional Maximum Loss $\mathrm{ML}$ for $L\in L_{exp}(\Omega ,{\mathcal {F}},P)$ by

9 $$\begin{aligned} \mathrm{ML}_{k}(L|\mathcal{{F}}') :=\sup \left\{ \mathbb {E}[LZ|\mathcal{{F}}']:\; Z\ge 0,\mathbb {E}[Z|\mathcal{{F}}']=1, {\mathbb {E}}[Z\log (Z)|\mathcal{{F}}']\le k\right\} . \end{aligned}$$

The $\sup$ here denotes the supremum of functions, with respect to almost sure ordering. The random variables *Z* can be interpreted as densities of feasible probability *Q* with respect to *P*. Furthermore (9) implies that $\mathrm{ML}$ is convex, homogeneous, and law-invariant.

If the losses $L({\mathbf {r}}_t)$ are independent of information ${\mathcal {F}}_0,\dots ,{\mathcal {F}}_{t-1}$ for all *t*, this is called the independence assumption in the following. Clearly, conditional risk measures as the conditional Maximum Loss are almost surely constant under the independence assumption, which is used in the following section.

Finally, we can apply the generic definitions (1) to a sequence of conditional Maximum Loss mappings, which leads to the multiperiod Maximum Loss, MML:

10 $$\begin{aligned} \mathrm{MML}_{\alpha _{T}}\left( L_{T}|\mathcal{{F}}_{T-1}\right)& := \mathrm{ML}_{\alpha _{T}} \left( L_{T}|\mathcal{{F}}_{T-1}\right) , \\ \mathrm{MML}_{\alpha _{t},\ldots ,\alpha _{T}} \left( L_{t}, \ldots , L_{T}|\mathcal{{F}}_{t-1}\right)& := \mathrm{ML}_{\alpha _{t}} \left( L_{t} + \mathrm{MML}_{\alpha _{t+1}, \ldots ,\alpha _{T}} \left( L_{t+1}, \ldots ,L_{T}|\mathcal{{F}}_{t-1}\right) \right) . \nonumber \end{aligned}$$

The notion of time unit invariance (2) then can also be applied to the multiperiod Maximum Loss. In fact, we will show in the following that $\mathrm{MML}$ is time unit invariant.

## Main results

Let us first look at the situation where a Kullback–Leibler radius $K>0$ for the long period [0, *T*] is given. Maximum cumulated loss over the whole period then is given by $\mathrm{ML}_{K}\left( \sum _{t=1}^T L_{t}\right)$. The following Lemma analyzes the connection between $\mathrm{ML}_K$ and the valuation by multiperiod Maximum Loss $\mathrm{MML}$, in particular the related one-period parameters $k_t$.

### **Lemma 1**

(Disaggregation) *Under the independence assumption, assuming (i) to (iii) for*$\Lambda (\theta , \sum _{t=1} ^{T}L_t)$*and a given*$K>0$*, the equation*

11 $$\begin{aligned} \theta \cdot \Lambda '\left( \theta , \sum _{t=1}^{T} L_t\right) - \Lambda \left( \theta , \sum _{t=1}^T L_t\right) =K \end{aligned}$$

*has a unique solution*${\overline{\theta }}$. *Choose*

12 $$\begin{aligned} k_{t}:={\overline{\theta }}\cdot \Lambda '({\overline{\theta }},L_{t})-\Lambda ({\overline{\theta }},L_{t}) \end{aligned}$$

*for*$t=1,\ldots ,T$. *With this choice we have*

13 $$\begin{aligned} \mathrm{MML}_{k_{1},\ldots ,k_{T}}(L_{1},\ldots ,L_{T}) = \mathrm{ML}_{K}\left( \sum _{t=1}^T L_{t}\right) , \end{aligned}$$

*and*

14 $$\begin{aligned} K=\sum _{t=1}^{T}k_{t}. \end{aligned}$$

### *Proof*

We prove this by induction in the number *T* of time steps. For $T=1$ the result is trivial. In what follows, choose ${\overline{\theta }}$ and the related $k_t$ according to the assumptions of the Lemma and Eq. (12). Summation leads to $\sum _{t=2}^T{k_t}={\overline{\theta }}\cdot \sum _{t=2}^T {\Lambda '({\overline{\theta }},L_{t})}-\sum _{t=2}^T{\Lambda ({\overline{\theta }},L_{t})}$. $\Lambda$ is the logarithm of a moment-generating function applied to a sum of independent random variables, hence

15 $$\begin{aligned} \sum _{t=2}^T{k_t}={\overline{\theta }}\cdot \Lambda ' \left( {\overline{\theta }},\sum _{t=2}^T{L_{t}}\right) -\Lambda \left( {\overline{\theta }},\sum _{t=2}^T{L_{t}}\right) . \end{aligned}$$

Assume now that the Lemma holds up to $T-1$ (induction hypothesis). Then–by renumbering—it holds also for losses $L_2,\dots , L_T$. Now, (15) shows that ${\overline{\theta }}$ and the $k_t$ are suitable for applying the Lemma to losses $L_2,\dots , L_T$ if one uses $\mathrm{ML}$ with radius $\sum _{t=2}^T k_t$. Hence we have

16 $$\begin{aligned} \mathrm{MML}_{k_{1},\ldots ,k_{T}} \left( L_{1},\ldots ,L_{T}\right) = \mathrm{ML}_{k_1}\left( L_1 +\mathrm{MML}_{k_{2},\ldots ,k_{T}} \left( L_{2},\ldots ,L_{T}\right) \right) \end{aligned}$$

17 $$\begin{aligned} \qquad \qquad \qquad \quad = \mathrm{ML}_{k_1}\left( L_1\right) +\mathrm{ML}_{\sum _{t=2}^T k_t}\left( \sum _{t=2}^T L_{t}\right) . \end{aligned}$$

By assumption and by (15) the relevant parameter is ${\overline{\theta }}$ for both $\mathrm{ML}$ in (17), therefore

18 $$\begin{aligned} \mathrm{MML}_{k_{1},\ldots ,k_{T}}(L_{1},\ldots ,L_{T})= \Lambda '\left( {\overline{\theta }},L_1\right) + \Lambda '\left( {\overline{\theta }},\sum _{t=2}^T L_t \right) \end{aligned}$$

19 $$\begin{aligned} \Lambda '\left( {\overline{\theta }},\sum _{t=1}^T L_t \right) = \mathrm{ML}_{K}\left( \sum _{t=1}^T L_t\right) , \end{aligned}$$

which shows (13). Finally, to establish (14) sum up Eq. (12) over *t*. $\square$

Now let us assume that positive real radii $k_{1},\ldots ,k_{T}$ for the individual periods are known. We give a characterization of the suitable parameter *K* for a valuation with *ML* over the full horizon.

### **Lemma 2**

(Aggregation) *Under the independence assumption and requiring (i–iii) for each given*$k_t$, *there exists a unique*${\overline{\theta }}$*such that*

20 $$\begin{aligned} \sum _{t=1}^{T}\Lambda ' \left( {\overline{\theta }},L_{t}\right) = \sum _{t=1}^{T}\Lambda ' \left( \theta _{t},L_{t}\right) , \end{aligned}$$

*where each*$\theta _{t}$*is the unique solution of*$\theta _{t} \cdot \Lambda '(\theta _{t},L_{t})- \Lambda (\theta _{t},L_{t}) = k_{t}$. *DefineKby*

21 $$\begin{aligned} K:={\overline{\theta }}\cdot \Lambda '\left( {\overline{\theta }},\sum _{t=1}^{T}L_{t}\right) -\Lambda \left( {\overline{\theta }},\sum _{t=1}^{T}L_{t}\right) . \end{aligned}$$

*With this choice ofKwe have*

22 $$\begin{aligned} \mathrm{ML}_{K}\left( \sum _{t=1}^{T}L_{t}\right) = \mathrm{MML}_{k_{1},\ldots ,k_{T}} \left( L_{1},\ldots ,L_{T}\right) =\sum _{t=1}^{T}\mathrm{ML}_{k_t}(L_t). \end{aligned}$$

### *Proof*

Since all $$\Lambda '(\cdot ,L_{t})$$ are strictly increasing and continuous, $$\Lambda '(\cdot ,\sum _{t=1}^{T} L_t)$$ is also strictly increasing and continuous, taking values in the interval $$[\Lambda '(\min (\theta _{t}),$$$$\sum _{t=1}^{T} L_t),\Lambda '(\max (\theta _{t}),\sum _{t=1}^{T} L_t)]$$. Therefore, for any point in this interval there is a unique $${\overline{\theta }}$$ in $$[\min (\theta _{t}),\max (\theta _{t})]$$ such that $$\Lambda '({\overline{\theta }},\sum _{t=1}^{T} L_t)$$ takes this value. Observing that under the independence assumption the right hand side of (20) equals $$\mathrm{MML}$$ and the left hand side is the $$\mathrm{ML}$$ of the summed loss variables with *K* given by (21), this establishes (22) and thus the lemma. $$\square$$

If in addition to the independence assumption the losses $L_t$ are identically distributed and $\alpha _t\equiv \alpha$, we can show that the properties of Definition 1 is fulfilled, i.e. Maximum Loss is time unit invariant.

### **Proposition 1**

(Time unit invariance) *Assume i.i.d. risk factors and a time independent loss functionL*, *which leads to i.i.d. losses*$L_t$. *Then under assumptions (i–iii):*

23 $$\begin{aligned} \mathrm{ML}_{kT}\left( \sum _{t=1}^{T}L_{t}\right) = \mathrm{MML}_{k,\ldots ,k} \left( L_{1},\ldots ,L_{T}\right) = T \cdot \mathrm{ML}_k(L). \end{aligned}$$

### *Proof*

Under the assumptions the losses $L_t$ are i.i.d. with the same distribution as some random variable *L*. By Lemma 2, there is unique $({\overline{\theta }},K)$ such that (22) holds. Putting ${\overline{\theta }}$ into (12) leads to $k_t=k$ and Lemma 1 gives $K=kT$. Equation (23) follows easily. $\square$

As required for time unit invariance, the one period parameter *k* leads to the multi-period parameter *kT*, independently of the actual loss function or of the risk factor distribution.

## Examples and counterexamples

In this section we discuss several examples and counterexamples for the property of time unit invariance. We start with an illustration of Proposition 1 by applying multiperiod Maximum Loss to linear and quadratic financial portfolios. After that, several other risk measures are discussed as building blocks for time consistent multiperiod risk measures. It turns out that multiperiod Maximum Loss is not the only time unit invariant risk measure: entropic risk measures (and as a special case the expectation) also has this property. On the other hand, two important risk measures, value at risk (which is not a risk measure in the strict sense, but widely used in industry) and expected shortfall (see e.g. Rockafellar and Uryasev 2000) are not time unit invariant, which is shown by counterexamples.

### Multiperiod Maximum Loss: linear portfolio, normal i.i.d. risk factors

Consider a non-constant linear portfolio over two periods, depending on one normal risk factor whose distributions at different times are independent. At each time t the risk factor takes a value $r_{t}\sim N(0,\sigma _{t}^{2})$. The loss of the portfolio at time *t* is $L_{t}(r_{t})=-l_{t}r_{t}$. One period unconditional Maximum Loss at radius $k_{t}$ can be calculated directly (see Breuer and Csiszár 2013 [Proposition 1]): $\Lambda (\theta ,L_{t})=\theta ^{2}\sigma _{t}^{2}l_{t}^{2}/2$, ${\overline{\theta }}=\sqrt{2k_{t}}/(\sigma _{t}|l_{t}|)$ and the Maximum Loss is $\Lambda '({\overline{\theta }},L_{t})=\sqrt{2k_{t}}\sigma _{t}|l_{t}|$. Two period Maximum Loss then is

$$\begin{aligned} \mathrm{MML}_{k_{1},k_{2}} \left( L_{1},L_{2}\right) = \sqrt{2k_{1}}\sigma _{1}|l_{1}| +\sqrt{2k_{2}}\sigma _{2}|l_{2}|. \end{aligned}$$

On the other hand Maximum Loss of the portfolio $L_{1}+L_{2}$ over the long period [0, 2] equals

$$\begin{aligned} \mathrm{ML}_{K} \left( L_{1}+L_{2}\right) = \sqrt{2K}\sqrt{\sigma _{1}^{2}l_{1}^{2}+ \sigma _{2}^{2}l_{2}^{2}}. \end{aligned}$$

The equality $\mathrm{ML}_{K}(L_{1}+L_{2})=\mathrm{MML}_{k_{1},k_{2}}(L_{1},L_{2})$ does not hold for $K=k_{1}+k_{2}$ but for

$$\begin{aligned} K=\frac{\left( \sqrt{k_{1}} \sigma _{1}|l_{1}|+\sqrt{k_{2}} \sigma _{2}|l_{2}|\right) ^{2}}{2\left( \sigma _{1}^{2} l_{1}^{2}+\sigma _{2}^{2}l_{2}^{2}\right) }. \end{aligned}$$

If, however, the portfolio is constant over the two time periods, $l_{1}=l_{2}$, the risk factor distributions at different times are identical, $\sigma _{1}=\sigma _{2}$, and the radii are equal, $k_{1}=k_{2}=:k$, then $K=2k$, in agreement with Proposition 1.

The same holds for *T* periods and if the portfolio loss at time *t* is given by a linear function of *n* risk factors, $L_{t}({\mathbf{r}}_{t})={\mathbf{l}}_{t}\cdot ({\varvec{\mu}}_{t}-{\mathbf{r}}_{t}),$ which are independent and normally distributed with mean ${\varvec{\mu}}_{t}$ and covariance matrix $\Sigma _{t}$, ${\mathbf {r}}_{t}\sim P_{t}=N({\varvec{\mu}}_{t},\Sigma _{t})$. In this case one period Maximum Loss for radius *k* equals $\sqrt{2k}\sqrt{{\mathbf {l}}_{t}^{T}\Sigma _{t}{\mathbf {l}}_{t}}$ (see Breuer and Csiszár 2013, [Proposition 2]). This implies

$$\begin{aligned} \mathrm{MML}_{k_{1},\ldots ,k_{T}}(L_{1},\ldots ,L_{T}) = \sum _{t=1}^{T} \sqrt{2k_{t}}\sqrt{{\mathbf {l}}_{t}^{T}\Sigma _{t}{\mathbf {l}}_{t}}. \end{aligned}$$

MaxLoss at radius *K* over one long period [0, *T*] of the aggregated cash flows equals

$$\begin{aligned} \mathrm{ML}_{K} \left( \sum _{t=1}^{T}L_{t}\right) =\sqrt{2K}\sqrt{\sum _{t=1}^{T} {\mathbf {l}}_{t}^{T}\Sigma _{t}{\mathbf {l}}_{t}}. \end{aligned}$$

The two are equal if

$$\begin{aligned} K=\frac{\left( \sum _{t=1}^{T}\sqrt{2k_{t}}\sqrt{{\mathbf {l}}_{t}^{T} \Sigma _{t}{\mathbf {l}}_{t}}\right) ^{2}}{2\sum _{t=1}^{T}{\mathbf {l}}_{t}^{T} \Sigma _{t}{\mathbf {l}}_{t}}. \end{aligned}$$

In general *K* is not equal to $\sum k_{t}$. But in the special case of a constant linear portfolio, ${\mathbf {l}}_{t}={\mathbf {l}}$, and iid normal risk factors, $\Sigma _{t}=\Sigma$ and $\mu _{t}=\mu$, we have equality if

$$\begin{aligned} K=\frac{ \left( \sum _{t=1}^{T}\sqrt{k_{t}}\right) ^{2}}{T}. \end{aligned}$$

If all $k_{t}$ are equal to *k*, then

$$\begin{aligned} K=Tk \end{aligned}$$

ensures the equality $\mathrm{ML}_{K}(\sum L_{t})=\mathrm{MML}_{k,\ldots ,k}(L_{1},\ldots ,L_{T})$, in agreement with Proposition 1. Maximum Loss at level *K* over the long period [0, *T*] equals multiperiod Maximum Loss, each at level *k*, if *K* grows linearly in *T*.

### Multiperiod entropic risk measures are time unit invariant

The loss expectation is time unit invariant in a trivial way: We have ${\mathbb {E}}(L_1+L_2)={\mathbb {E}}(L_1+{\mathbb {E}}(L_2)|{\mathcal {F}}_1)$, which is true for any specification of $L_1,L_2$ and ${\mathcal {F}}$ and hence also holds for i.i.d. variables.

We will see now that time unit invariance can be shown for a larger family of risk measures, which contains the expectation as a special case.

Entropic acceptability functionals $ENT_{\gamma }$ with parameter $\gamma >0$ can be defined for losses *L* by

24 $$\begin{aligned} ENT_{\gamma }\left[ L\right] =\frac{1}{\gamma }\cdot \log \left( {\mathbb {E}} \left[ e^{\gamma \cdot L}\right] \right) =\frac{1}{\gamma }\cdot \Lambda \left( \gamma ,L\right) \end{aligned}$$

with continuous extension $ENT_{0}\left[ L\right] ={\mathbb {E}}[L]$. Hence, the expectation is a special case—the entropic risk measure with $\gamma =0$. Here $\Lambda (\cdot ,L)$ again denotes the cumulant generating function of *L*. Conditional entropic measures then can be introduced as

25 $$\begin{aligned} ENT_{\gamma }\left[ L\right| {\mathcal {F}}]=\frac{1}{\gamma }\cdot \log \left( {\mathbb {E}}\left[ e^{\gamma \cdot L}\right] |{\mathcal {F}}\right) \end{aligned}$$

with $ENT_{0}\left[ L|{\mathcal {F}}\right] ={\mathbb {E}}[L|{\mathcal {F}}]$. Entropic risk functionals were introduced in Föllmer and Schied (2002) and further studied in e.g. Detlefsen and Scandolo (2005). Note that usually they are defined in terms of gains $X=-L$, instead of losses. It has been shown in Kupper and Schachermayer (2009) that the only convex, law invariant, time consistent, “relevant” risk measures for processes are the entropic risk measures.

Conditional entropic risk measures can be concatenated according to (1), which leads to a multiperiod risk entropic risk measure, $MENT_{\gamma _1,\dots ,\gamma _T}$.

It turns out that multiperiod entropic risk measures $MENT_{\gamma ,\dots ,\gamma }$ are time unit invariant. A full proof, using the properties of cumulant generating functions $\Lambda$, again can be furnished by induction. In fact, by definitions (25) and (24), this is even simpler than in the case of Maximum Loss. For simplicity, we demonstrate here the case of two losses.

Consider $L_1,L_2$, two i.i.d. losses at time 1 and 2, and the (conditional) entropic risk measure with parameter $\gamma$. The multiperiod entropic risk measure then is given by

26 $$\begin{aligned} MENT_{\gamma ,\gamma }\left( L_{1},L_{2}\right)& = ENT_{\gamma }\left( L_{1} +ENT_{\gamma }\left( L_{2}\right) \right) \nonumber \\& = \frac{1}{\gamma }\Lambda \left( \gamma ,L_{1}+\frac{1}{\gamma } \Lambda \left( \gamma ,L_{2}\right) \right) \nonumber \\& = \frac{1}{\gamma }\Lambda \left( \gamma ,L_{1}\right) +\frac{1}{\gamma } \Lambda \left( \gamma ,L_{2}\right) \nonumber \\& = \frac{2}{\gamma }\Lambda \left( \gamma ,L_{1}\right) , \end{aligned}$$

where the third equality is implied by the general fact $\log \left( {\mathbb {E}}\left[ e^{\gamma X+a}\right] \right) =\log \left( {\mathbb {E}}\left[ e^{\gamma X}\right] \right) +\gamma a$, which holds for any suitable random variable *X* and real number *a*.

On the other hand we have

27 $$\begin{aligned} ENT_{\gamma }\left( L_{1}+L_{2}\right)&= \frac{1}{\gamma } \Lambda \left( L_{1}+L_{2}\right) \nonumber \\ & = \frac{1}{\gamma }\Lambda \left( \gamma ,L_{1}\right) + \frac{1}{\gamma }\Lambda \left( \gamma ,L_{2}\right) \nonumber \\ &= \frac{2}{\gamma }\Lambda \left( \gamma ,L_{1}\right) . \end{aligned}$$

Altogether we have

28 $$\begin{aligned} MENT_{\gamma ,\gamma }\left( L_{1},L_{2}\right) =ENT_{\gamma }\left( L_{1}+L_{2}\right) , \end{aligned}$$

which holds independently of the loss distribution. This ensures time unit invariance. Compared to Maximum Loss the parameter over both periods actually equals the parameter over the shorter periods.

### Multiperiod value at risk is not time unit invariant

For a constant linear portfolio with loss function $L_{t}(r_{t})=-lr_{t}$ whose loss equals a constant $-l$ times the value of a normally distributed risk factor $r_t\sim N(0,\sigma ^2)$. Value at Risk at level $\alpha$ equals $\Phi ^{-1}(\alpha )\cdot \sigma \cdot l$, where $\Phi ^{-1}$ is the inverse of the standard normal distribution function. For a sequence of confidence levels $\alpha _1,\alpha _2,\ldots ,\alpha _T$, a time consistent multiperiod version $\mathrm{MVaR}$ of VaR may be defined by a procedure similar to (1) with $\mathrm{VaR}_\alpha$ in the role of $\mathrm{R}_\alpha$. For two time steps we get

$$\begin{aligned} \mathrm{MVaR}_{\alpha _1,\alpha _2} \left( -lr_{1},-lr_{2}\right) = \left( \Phi ^{-1}\left( \alpha _1\right) + \Phi ^{-1}\left( \alpha _2\right) \right) \cdot \sigma \cdot l. \end{aligned}$$

Aggregated VaR at level $\bar{\alpha }$ over the period [0, 2] equals

$$\begin{aligned} \mathrm{VaR}_{\bar{\alpha }}\left( -lr_{1}-lr_{2}\right) = \sqrt{2} \Phi ^{-1}\left( \bar{\alpha }\right) \cdot \sigma \cdot l. \end{aligned}$$

$\mathrm{MVaR}_{\alpha ,\alpha }$ equals one period VaR at level $\bar{\alpha }$ if

29 $$\begin{aligned} \bar{\alpha }=\Phi \left( \left( \Phi ^{-1}\left( \alpha _1\right) + \Phi ^{-1}\left( \alpha _2\right) \right) /\sqrt{2}\right) . \end{aligned}$$

This condition guarantees equality for all constant linear portfolios.

Consider now quadratic portfolios with loss function $-l^{2}r^{2}$. $\mathrm{VaR}_\alpha$ equals $F_1^{-1}(\alpha )l^{2}\sigma ^{2}$, where $F_1^{-1}$ is the inverse of the distribution function of the $\chi ^{2}$-distribution with one degree of freedom. Multiperiod VaR for the parameter sequence $(\alpha _{1},\alpha _{2})$ is

$$\begin{aligned} \mathrm{MVaR}_{\alpha _{1},\alpha _{2}} \left( -l^{2}r_{1}^{2}, -l^{2}r_{2}^{2}\right) = \left( F_1^{-1}\left( \alpha _{1}\right) + F_1^{-1}\left( \alpha _{2}\right) \right) \sigma ^{2}l^{2}. \end{aligned}$$

Aggregated VaR at level $\bar{\alpha }$ over the period [0, 2] is

$$\begin{aligned} \mathrm{VaR}_{\bar{\alpha }}\left( -l^{2}r_{1}^{2}-l^{2}r_{2}^{2}\right) = F_2^{-1}\left( \bar{\alpha }\right) l^{2}\sigma ^{2}, \end{aligned}$$

where $F_2^{-1}$ is the inverse of the distribution function of the $\chi ^{2}$-distribution with two degrees of freedom. Multiperiod $\mathrm{MVaR}_{\alpha _1,\alpha _2}(-l^{2}r_{1}^{2},-l^{2}r_{2}^{2})$ equals $\mathrm{VaR}_{\bar{\alpha }}(-l^{2}r_{1}^{2}-l^{2}r_{2}^{2})$ if

30 $$\begin{aligned} \bar{\alpha }=F_{2}\left( F_1^{-1}\left( \alpha _1\right) + F_1^{-1}\left( \alpha _2\right) \right) . \end{aligned}$$

This guarantees equality for all constant quadratic portfolios. But (30) is different from (29), hence time unit invariance is not fulfilled for MVaR.

### Multiperiod expected shortfall is not time unit invariant

Assume that losses $L_{i}$ are i.i.d. with a standard normal distribution. (Conditional) expected shortfall of $L_{2}$ at level *q* therefore is

$$\begin{aligned} ES_{1}\left[ L_{2}\right] =ES\left[ L_{2}\right] = \frac{\phi \left( \Phi ^{-1}(q) \right) }{1-q}. \end{aligned}$$

The multiperiod expected shortfall over two periods then is given by

$$\begin{aligned} ESM(q)=ES\left[ L_{1}+ES_{1}\left[ L_{2}\right] \right] =ES\left[ L_{1}\right] + \frac{\phi \left( \Phi ^{-1}(q)\right) }{1-q}=2\cdot \frac{\phi \left( \Phi ^{-1} (q)\right) }{1-q}. \end{aligned}$$

On the other hand the expected shortfall over two periods at level $q^{\prime }$ is given by the expected shortfall of the random variable $L_{1}+L_{2}$, which has a normal distribution with mean zero and a variance of two. This leads to

$$\begin{aligned} ESS(q^{\prime })=\sqrt{2}\cdot \frac{\phi \left( \Phi ^{-1}(q^{\prime })\right) }{1-q^{\prime }}. \end{aligned}$$

To find a level $q^{\prime }$ such that ESM and ESS are equal, one has to solve

31 $$\begin{aligned} \frac{\phi \left( \Phi ^{-1}(q^{\prime })\right) }{1-q^{\prime }}= \sqrt{2}\cdot \frac{\phi \left( \Phi ^{-1}(q)\right) }{1-q}. \end{aligned}$$

Consider now the quadratic portfolio: $M_{i}=L_{i}^{2}$ are i.i.d. $\chi ^{2}$-distributed with one degree of freedom. Here we have

$$\begin{aligned} ES_{1}\left[ M_{2}\right] =ES\left[ M_{2}\right] =1+\sqrt{2} \frac{\sqrt{F_{1}^{-1}(q)\exp \left( -\frac{1}{2}F_{1}^{-1}(q)\right) }}{1-q}, \end{aligned}$$

and

$$\begin{aligned} ESM(q)=ES\left[ M_{1}+ES_{1}\left[ M_{2}\right] \right] =2+2\sqrt{2} \frac{\sqrt{F_{1}^{-1}(q)\exp \left( -\frac{1}{2}F_{1}^{-1}(q)\right) }}{1-q}, \end{aligned}$$

where $F_{1}^{-1}(q)$ denotes the *q*-quantile of the $\chi ^{2}$ distribution with one degree of freedom.

The expected shortfall over two periods is calculated for $M_{1}+M_{2}$, which is $\chi ^{2}$-distributed with two degrees of freedom:

$$\begin{aligned} ESS(q^{\prime })=\frac{\exp \left( -\frac{1}{2}F_{2}^{-1}(q^{\prime }) \right) \cdot \left( F_{2}^{-1}(q)+2\right) }{1-q}, \end{aligned}$$

where $F_{2}^{-1}(q)$ denotes the *q*-quantile of the $\chi ^{2}$ distribution with two degrees of freedom.Again we have to solve ESM(q) = ESS$(q^{\prime })$. Because of

$$\begin{aligned} F_{2}^{-1}(q^{\prime })=-2\ln \left( 1-q^{\prime }\right) , \end{aligned}$$

this leads to

$$\begin{aligned} q^{\prime }=1-\exp \left( \frac{1}{2}+\frac{\sqrt{2}}{2} \frac{\sqrt{F_{1}^{-1}(q)\exp \left( -\frac{1}{2}F_{1}^{-1}(q)\right) }}{1-q}\right) , \end{aligned}$$

which is not in general a solution to (31).

## Conclusions

We introduced the process risk measure $\mathrm{MML}$ and analyzed under which conditions it equals one period Maximum Loss of the aggregated losses. Assuming losses at time *t* are independent of information at time $t-1$ we arrived at the following results.

(1) Given a radius *K* for the maximum aggregated losses over [0, *T*], it is possible to find radii $k_t$ for each time step, such that $\mathrm{MML}$ equals one period Maximum Loss with the given radius *K*. Here, $\sum k_{t}=K$ holds.

(2) Given radii $k_{t}$ for each *t*, it is possible to calculate an overall radius *K* such that $\mathrm{MML}$ equals the one period maximum aggregated loss at radius *K*. Under this conditions $\sum k_{t}=K$ does not hold in general.

(3) If in situation (2) risk factors are identically distributed and the portfolio composition does not change, the relation $k_{t}=\frac{K}{n}$ holds for all distributions of the $r_{t}$ and for all loss functions *L*. This is invariance under changes of time units as defined in Definition 1.

In addition it turned out that concatenations of entropic risk measures are also time unit invariant. In particular the expectation is time unit invariant. On the other hand, time consistent multiperiod versions of Value at Risk and do not own this property. The same is true for concatenations of Expected Shortfall.
